# Guselkumab‐associated bullous pemphigoid in a psoriasis patient: A case report and review of the literature

**DOI:** 10.1111/dth.15207

**Published:** 2021-11-30

**Authors:** Martina Burlando, Niccolò Capurro, Astrid Herzum, Emanuele Cozzani, Aurora Parodi

**Affiliations:** ^1^ Department of Health and Science (Dissal), Section of Dermatology University of Genoa, Polyclinic Hospital San Martino, IRCCS Genoa Italy

**Keywords:** adverse reaction, biologic therapy, drug pemphigoid, guselkumab, psoriasis

## Abstract

Drug‐induced bullous pemphigoid (DBP) associated to biologics administered for psoriasis is rare. DBP has been described especially in association with anti‐TNF‐α drugs and anti‐IL12 and 23, but never in relation to guselkumab (anti‐IL23). We report the case of a 76‐year‐old male patient with severe psoriasis (PASI 20), presenting with generalized tense bullae and erosions after being recently switched to guselkumab therapy. Histology and direct immunofluorescence confirmed the suspect of bullous pemphigoid (BP). Guselkumab administration was interrupted, low‐dose oral corticosteroid therapy was introduced and after only 1‐month remission was obtained with no new lesions appearing. As outlined in the presented case, DBP's onset typically follows the introduction of a new drug in patients taking polypharmacy. In addition, DBP may spontaneously regress after discontinuation of the triggering drug and it responds very rapidly to steroid therapy. Up to date, DBP has been described after biological therapy for psoriasis in 11 patients, following administration of ustekinumab, efalizumab, etanercept, secukinumab, and adalimumab. Conversely, DBP after guselkumab therapy for psoriasis has never been reported in published studies. We highlight the need to face and document increasing, though rare, side effects of biologic therapies, as new biologic molecules are being constantly developed and administered to psoriatic patients, to promptly interrupt treatment when needed.

## 1 INTRODUCTION

Biologic drugs have become a pivotal treatment for moderate‐to‐severe psoriasis, as they are effective, easy‐to‐administer, and safe.^1^ Indeed, adverse drug reactions are mostly mild, including flu‐like symptoms and minor dermatological manifestations, such as injection site reactions. Rarer cutaneous adverse drug reactions include drug‐induced bullous pemphigoid (DBP), which has been reported in connection with the administration of some biologic drugs, especially anti‐TNF‐α drugs and anti‐IL12 and 23, but never with regard to guselkumab (anti‐IL23).^2^ To the best of our knowledge, this is the first reported case of guselkumab‐induced bullous pemphigoid in the literature. Written informed consent was obtained from the patient for publication of this case report and any accompanying images.

## 2 CASE REPORT

We report the case of a 76‐year‐old male patient with severe psoriasis (PASI 20), presenting at the dermatology clinic for the onset of multiple bullae on the legs, 4 weeks after switching to guselkumab from ustekinumab due to loss of efficacy.

The patient, who was also on chronic therapy with multiple drugs for diabetes mellitus, chronic heart failure, and atrial fibrillation, reported no prior significant side effects, and routine blood tests were normal.

**FIGURE 1:**
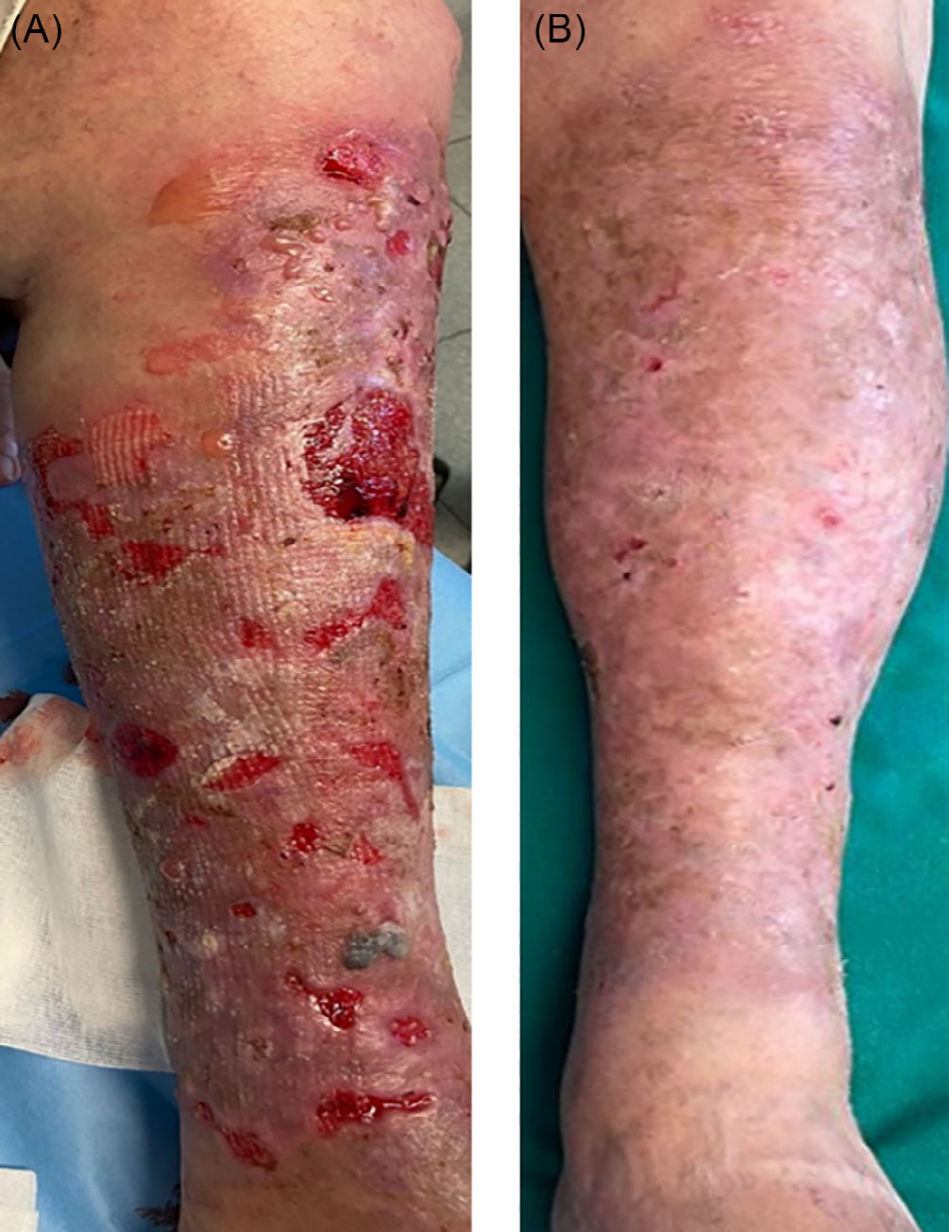
(A) Before treatment, clinical presentation of BP with extended tense bullae on the legs and crusted erosions. (B) After 4 months of drug interruption and steroid therapy crusty outcomes of resolving bullae and scarring

At physical examination, extended tense bullae and crusted erosions, on oedematous‐erythematous skin, were observed on his lower limbs (Figure 1A).

**FIGURE 2:**
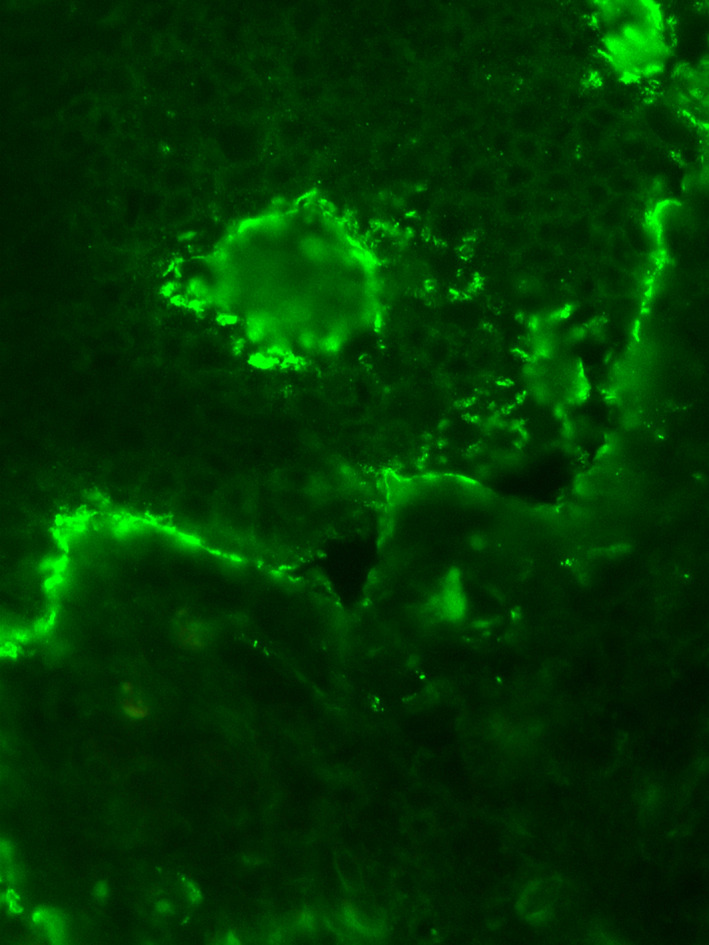
Direct immunofluorescence (DIF) showing typical BP features: linear IgG and C3 deposits at the dermoepidermal junction, and numerous IgA and IgM in the dermis

Histology confirmed epidermal separation from the dermoepidermal junction, with intense eosinophilic infiltrate. Direct immunofluorescence revealed linear IgG and C3 deposits at the dermoepidermal junction, and abundant IgA and IgM in the dermis (Figure 2).

Enzyme‐linked immunosorbent assay test showed positive anti‐BP180 and anti‐BP230 antibodies confirming the clinical suspicion of DBP.

Guselkumab administration was withdrawn, and low‐dose oral corticosteroid therapy (prednisone 25 mg/die) was started, achieving rapid clinical improvement in 1 week. Remission was achieved after 1 month and no new lesions appeared. Prednisone was then tapered to 10 mg/die over 3 months and still no new lesions appeared. Only extensive crusts of resolving bullae were seen, confirming the hypothesis of DBP (Figure 1B).

## 3 DISCUSSION

Drug‐associated bullous pemphigoid (DBP) presents with distinctive features, distinguishing it from the idiopathic form of bullous pemphigoid (IBP). Typically, acute onset DBP follows the administration of a new drug, usually in patients who are already receiving polypharmacy, as in our case.^2^

Histologically, DBP is characterized by a rich eosinophilic infiltrate at the dermal‐epidermal junction, as can be observed in our patient, while immunofluorescence reveals positivity to the same antigens as IBP.^2^

In addition, DBP responds very rapidly to steroid therapy, which may not even be necessary given the rapid spontaneous resolution after discontinuation of the triggering drug.^2^

In our case, we considered it appropriate to introduce also oral steroid therapy, given the extension of the lesions, though the patient's clinical picture rapidly improved after discontinuation of the triggering drug and complete remission was obtained just 1 month after the drug discontinuation and low‐dose steroid therapy.

It has been widely demonstrated that pharmacological agents, such as nonsteroid inflammatory drugs, PD1‐PDL‐1 inhibitors, diuretics, and penicillin are likely causally implicated in DBP.

Biologics are increasingly being reported as triggers of DBP.

DBP in psoriasis patients treated with biologics has been reported after the administration of ustekinumab in five patients, after efalizumab in two, after etanercept in two, after secukinumab in one, and after adalimumab in one patient.^3^, ^4^, ^5^, ^6^, ^7^, ^8^, ^9^, ^10^, ^11^, ^12^, ^13^, ^14^, ^15^ Conversely, DBP following guselkumab biologic therapy for psoriasis has never been reported.

It is well known that biologic therapies target final pathogenetic pathways of psoriasis and selectively inhibit Th1 and Th17 clonal expression and differentiation, thereby reducing the formation of proinflammatory cytokines.^1^, ^5^ It has been suggested that alteration of cytokine pathways causing cytokine imbalance may play a role as immunopathogenic mechanism of DBP, though specific pathogenetic mechanisms are not yet understood.^5^ Future studies are warranted to help deepen this issue.

As new biologic molecules are being constantly developed and administered to psoriatic patients, dermatologist are called to face increasing, though rare, side effects and promptly interrupt treatment when needed.^1^

## CONFLICT OF INTEREST

The authors have no conflicts of interest to declare.

## AUTHOR CONTRIBUTIONS

All authors approved the final version of the manuscript. **Martina Burlando**: Drafted the work and revised it critically for important intellectual content, gave final approval of the version to be published, agreed to be accountable for all aspects of the work. **Niccolò Capurro**: Drafted the work and revised it critically for important intellectual content, gave final approval of the version to be published, agreed to be accountable for all aspects of the work. **Astrid Herzum**: Drafted the work and revised it critically for important intellectual content, gave final approval of the version to be published, agreed to be accountable for all aspects of the work. **Emanuele Cozzani**: Revised the work critically for important intellectual content, gave final approval of the version to be published, agreed to be accountable for all aspects of the work. **Aurora Parodi**: Revised the work critically for important intellectual content, gave final approval of the version to be published, agreed to be accountable for all aspects of the work. The authors thank Paola Canepa and Simonetta Verdiani for their relevant contribution to the immunofluorescence studies.

## Data Availability

The data that support the findings of this study are available from the corresponding author upon reasonable request.
